# Residual setup errors in cranial stereotactic radiosurgery without six degree of freedom robotic couch: Frameless versus rigid immobilization systems

**DOI:** 10.1002/acm2.12828

**Published:** 2020-02-18

**Authors:** Raymond J. Liu, Scarlet X. Yang, John Neylon, Matthew D. Hall, Savita Dandapani, Nayana Vora, Jeffrey Y. C. Wong, An Liu

**Affiliations:** ^1^ City of Hope National Medical Center Duarte CA USA; ^2^ Miami Cancer Institute Miami FL USA

**Keywords:** cranial stereotactic radiosurgery, immobilization, margin, Aktina, TALON, six DoF robotic couch

## Abstract

**Purpose and Objectives:**

This IRB‐approved study was to compare the residual inter‐fractional setup errors and intra‐fractional motion of patients treated with cranial stereotactic radiosurgery without a 6 degree of freedom (DoF) couch. We evaluated both frameless non‐invasive vacuum‐suction immobilization (Aktina PinPoint) and TALON rigid screw immobilization.

**Materials and Methods:**

Twenty consecutive patients treated by Varian TrueBeam STX or Tomotherapy were selected for data collection. The dose and number of fractions received by each patient ranged from 18 Gy in 1 fraction (SRS) to 25 Gy in 5 fractions (SRT). Twelve patients were immobilized using PinPoint, a frameless suction system (Aktina Medical, New York) and eight patients were immobilized using the TALON rigid screw system. Customized head cushions were used for all patients. Six Atkina patients received pre‐ and post‐treatment cone‐beam CT (CBCT) to evaluate the intra‐fractional motion of the Aktina system. The intra‐fractional motion with the TALON rigid screw system has been reported to be negligible and was not repeated in this study. All patients received pre‐treatment CBCT or megavoltage CT (MVCT) to assess inter‐fractional setup accuracy. Shifts to the final treatment position were determined based on matching bony anatomy in the pre‐treatment setup CT and the planning CT. Setup CT and planning CT were registered retrospectively based on bony anatomy using image registration software to quantify rotational and translational errors.

**Results:**

For the frameless Aktina system, mean and standard deviation of the intra‐fractional motion were −0.5 ± 0.7 mm (lateral), 0.1 ± 0.9 mm (vertical), −0.5 ± 0.6 mm (longitudinal), −0.04 ± 0.18°(pitch), −0.1 ± 0.23°(yaw), and −0.03 ± 0.17°(roll) indicating negligible intra‐fractional motion. Inter‐fractional rotation errors were −0.10 ± 0.25° (pitch), −0.08 ± 0.16° (yaw), and −0.20 ± 0.41° (roll) for TALON rigid screw immobilization versus 0.20 ± 0.69° (pitch), 0.34 ± 0.56° (yaw), 0.35 ± 0.82° (roll) for frameless vacuum‐suction immobilization showing that the rigid immobilization setup is more reproducible than the frameless immobilization. Without rotational correction by a 6 DoF couch, residual registration error exists and increases with distance from the image fusion center. In a 3D vector space, a tumor located 5 cm from the center of image fusion would require a 0.9 mm margin with the TALON system and a 2.1 mm margin with Aktina.

**Conclusions:**

With image‐guided radiotherapy, translational setup errors can be corrected by image registration between pre‐treatment setup CT and planning CT. However, rotational errors cannot be accounted for without a 6 DoF couch. Our study showed that the frameless Aktina immobilization system provided negligible intra‐fractional motion. The inter‐fractional rotation setup error using Aktina was larger than rigid immobilization with the TALON system. To treat a single lesion far from the center of image registration or for multiple lesions in a single plan, additional margin may be needed to account for the uncorrectable rotational setup errors.

## 1 INTRODUCTION

Immobilization devices are commonly used in radiation therapy as they provide reproducible patient positioning. For intracranial lesions, treatment positioning, and immobilization that provide sub‐millimeter accuracy is desired for single fraction stereotactic radiosurgery (SRS) or fractionated stereotactic radiotherapy (SRT).^1^, ^2^

The positioning accuracy includes inter‐fraction setup reproducibility (localization) and intra‐fraction motion (fixation). Inter‐fraction error relates to the setup accuracy of the same posture and position between CT simulation and treatment delivery or subsequent deliveries (SRT). The intra‐fraction error is the amount of patient motion over the course of a single treatment. There are many commercially available immobilization devices designed to reduce intra‐fraction and inter‐fraction motion in intracranial radiation treatment. Invasive devices include the Leksell,^3^, ^4^ Reichert‐Mundinger,^5^ and Brown‐Roberts‐Wells (BRW)^6^ frames. Traditionally, those invasive head frames are attached to the patient during the entire planning and treatment course. It is believed the device can fix the cranium rigidly for perfectly reproducible patient geometry. Thus no treatment margin beyond the treated target is needed.^2^, ^3^, ^4^, ^5^, ^6^, ^7^ Non‐invasive options use one of or a combination of thermoplastic masks, bite blocks, cradles, and optical surface tracking imaging systems to localize and immobilize the brain.^8^ While the invasive devices can often offer sub‐millimeter positional accuracy, the invasive nature limits their acceptance by patients and deters fractionated treatments. In the non‐invasive realm, vendors are constantly developing new techniques to improve the positioning accuracy of their devices.

In this study, we compared two commercially available devices used in our clinic: Aktina PinPoint™ (non‐invasive)^8^ and TALON Cranial SRS frame (invasive).^9^ Both intra‐fraction and inter‐fraction setup errors were evaluated. Additionally, recommendations on margins required to compensate for residual set up errors were discussed.

## 2 METHOD AND MATERIALS

Twenty consecutive patients treated by Varian TrueBeam STX or Tomotherapy were selected for data collection and analysis.

### 2.1 TALON rigid immobilization

**Figure 1:**
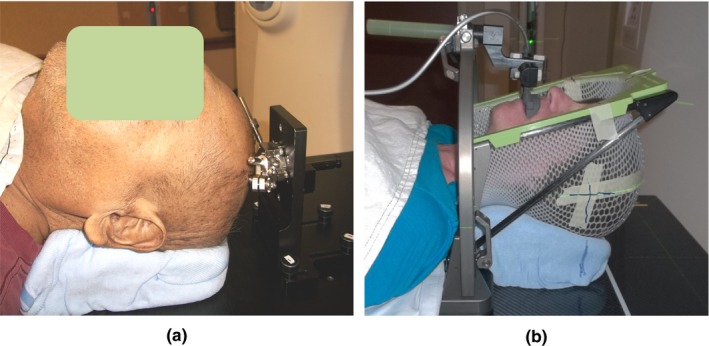
Invasive TALON (a) immobilization device and noninvasive Aktina (b) device for intracranial lesions.

The TALON system was originally developed by Nomos Corp (now part of Best Medical International, Inc, Springfield, Virginia) as a less invasive alternative to frame‐based SRS immobilization devices [Fig. 1(a)]. Neurosurgeon implants two titanium base screws into the patient's skull. It can be performed under local anesthesia in an outpatient surgical suite. TALON system has many adjustable locking ball joints and extension rods. During CT simulation, the customizable TALON system is attached to the base screws. The joints and rods are adjusted to fit patient specific anatomy and screw locations. Once in position, all degrees of freedom of the device are tightened and locked. TALON is fastened to the base plate which is attached to couch, preventing movement of the patient's head. The TALON device provides a patient‐specific rigid immobilization of the head. The particular TALON is used throughout the individual patient's treatment course, SRS or SRT. Between CT simulation and treatment, it is detached from the screws in the patient's skull but the integrity of the ball joints and geometry remains untouched. Essentially, the TALON system is a removable stereotactic immobilization system using a one‐time invasive placement of two titanium screws and a noninvasive daily application.

### 2.2 Aktina frameless immobilization

The Aktina PinPoint (Aktina Medical, Congers, New York) is a non‐invasive frameless alternative designed for intracranial single fraction and multi‐fraction SRS treatments [Fig. 1(b)]. This device contains a patient‐specific mouth piece which is constructed during CT simulation to fit the individual patient's dental anatomy. After the mold is made, vacuum suction between the mold and the upper hard palate establishes a firm seal. The mouth piece is attached to the couch via Aktina metal frame to prevent motion in any direction. Together with a customized head cushion, the Aktina helps to position the patient's head in the same geometry and renders cranium fixation during the course of treatment. Because of its noninvasive nature, the usage of Aktina has potential advantage over any invasive device including TALON.

### 2.3 CT simulation

**Figure 2:**
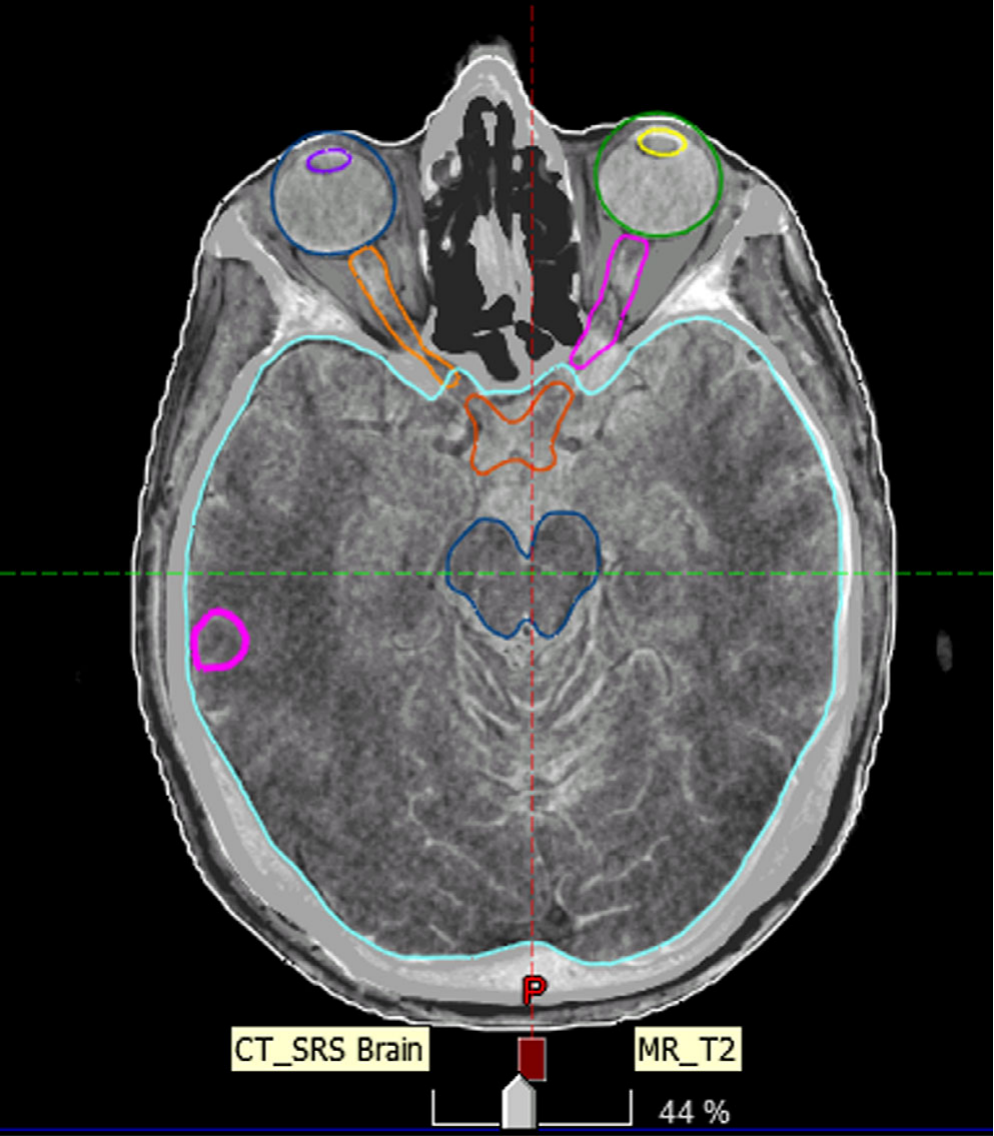
Registered CT and MR images were used to contour the normal structures and targets.

All 20 patients analyzed in this study were CT‐simulated using the GE Discovery 590RT™ 16 slice large bore CT scanner. Patient was either immobilized with TALON (8 patients) or Aktina (12 patients) in conjunction with Accuform™ (CIVCO, Kalona, Iowa) head cushion. The same scanning technique (120 KV, 200 mA, 512 × 512 image size with 1.25 mm slices and spacing) was used. Images were transferred to Eclipse (Varian, Palo Alto, CA) treatment planning computer. The CT data were registered in Eclipse using rigid registration with MRI for contouring. Typical organ and target contours included GTV, PTV, brain stem, lens, eyes, optic nerves, optic chiasm, and brain. Figure 2 shows the regions of interest on registered CT and MR images.

### 2.4 Treatment and data collection

**Figure 3:**
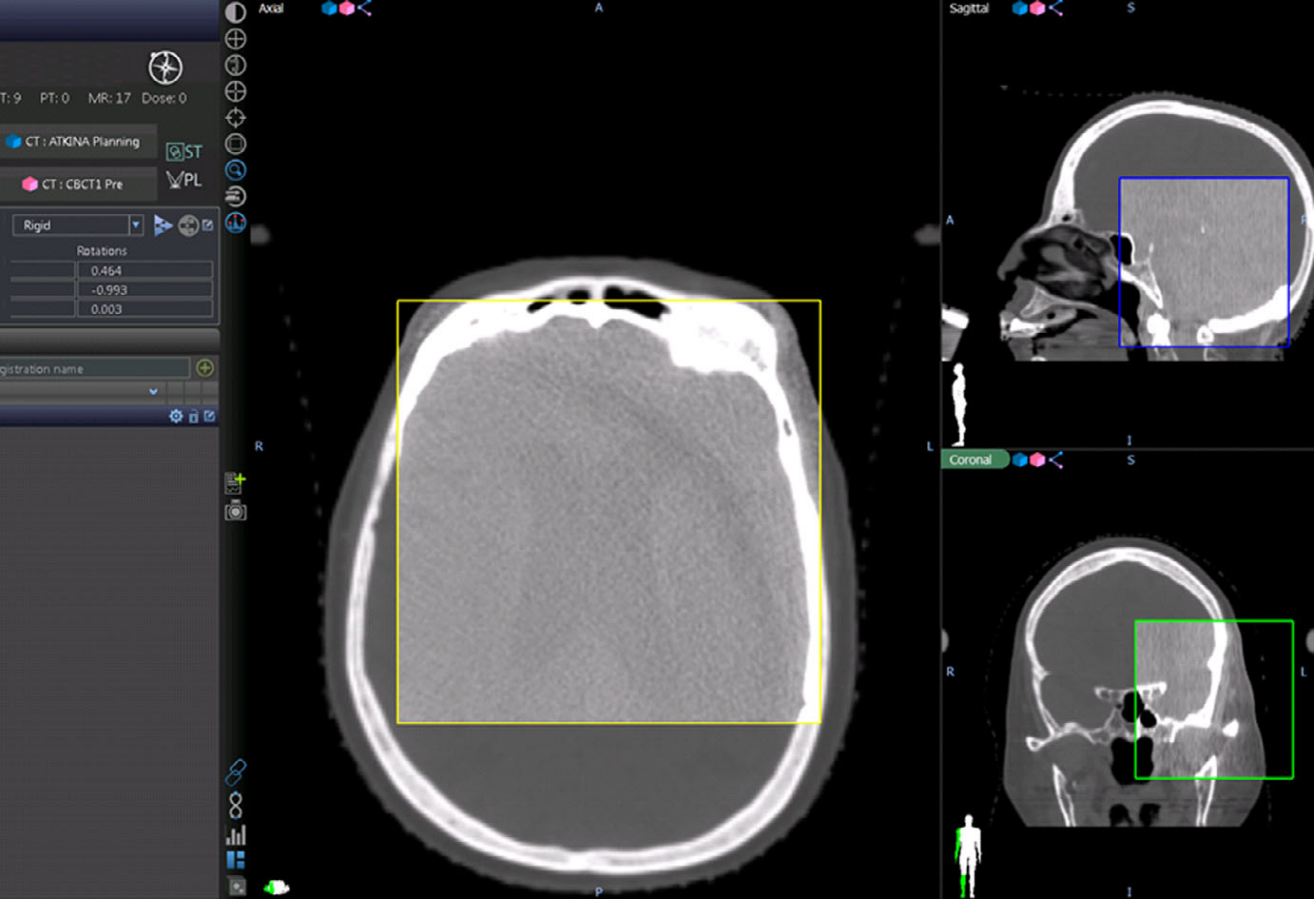
Auto registration between simulation planning CT (primary image) and pre‐treatment CBCT (inside the box) using whole brain bony anatomy.

The dose and number of fractions received by each patient ranged from 18 Gy in 1 fraction (SRS) to 25 Gy in 5 fractions (SRT). Two patients received single fraction SRS and the remaining 18 received 3 to 5 fraction SRT. All patients received pre‐treatment CBCT on TrueBeam STX or megavoltage CT (MVCT) on Tomotherapy to assess inter‐fractional setup accuracy. The six Aktina patients treated on a TrueBeam STX and also received post‐treatment cone beam CT (CBCT) to evaluate the intrafractional motion of the Aktina system. Six Aktina patients and eight TALON patients were treated on a Tomotherapy machine. The pre‐treatment setup CT and the planning CT Images were auto‐registered using vendor provided image registration software based on bony anatomy (Fig. 3). Attending physicians reviewed and approved every image registration before treatment. Shifts to the final treatment position were documented. Because this study was conducted before the 6 degrees of freedom robotic couch was installed, not all rotational (pitch, roll, and yaw) correction could be performed for the actual treatments.

### 2.5 Image fusion

With a 6 DoF robotic couch, all shifts (three translational and three rotational) can be corrected. If the brain is a perfect rigid body and there is no image geometric distortion, the pre‐treatment setup CT and the planning CT Images registration can perfectly reproduce the treatment position to the same as the simulation one. In a real patient, image acquisition techniques, image registration algorithm, and image distortion due to physiological changes can all compromise the image fusion and thus the treatment accuracy.

**Figure 4:**
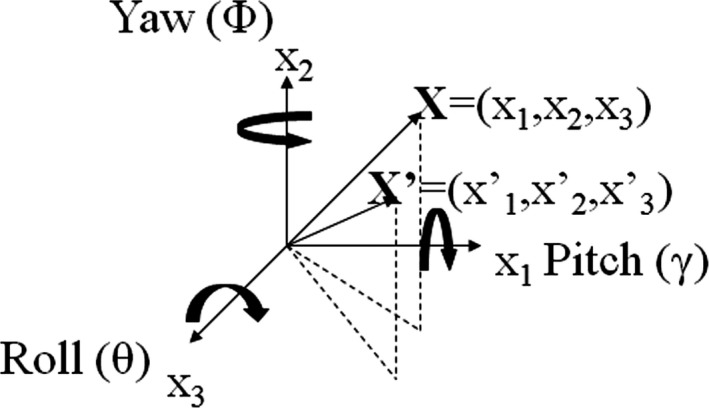
Three‐dimensional rotational corrections.

Assuming points X and X′ are the same anatomy points in 2 image datasets (Fig. 4), the misalignment can be corrected by formula (1).

(1) $$X^{\prime }=P\left(\gamma \right)R\left(\theta \right)Y\left(\phi \right)\text{X,}$$

where$P=\left(\begin{array}{ccc} 1 & 0 & 0\\ 0 & \cos\;\gamma & \sin\;\gamma \\ 0 & -\sin\;\gamma & \cos\;\gamma \end{array}\right)$ is the correction for pitch rotational error, $R=\left(\begin{array}{ccc} \cos\;\theta & \sin\;\theta & 0\\ -\sin\;\theta & \cos\;\theta & 0\\ 0 & 0 & 1 \end{array}\right)$, and $Y=\left(\begin{array}{ccc} \cos\;\phi & 0 & \sin\;\phi \\ 0 & 1 & 0\\ -\sin\;\phi & 0 & \cos\;\phi \end{array}\right)$ are the corrections for roll and yaw.

Please note, the sequence applying the correction matters. This formulation is correct only if yaw is performed first, then the roll and finally the pitch.

Formula (1) was used to estimate the systemic errors introduced when 6 degrees of freedom robotic couch is not available and rotational errors cannot be corrected.

## 3 RESULTS

### 3.1 Intra‐fraction motion

Intra‐fraction motion under Aktina immobilizations analyzed with pre‐ and post‐treatment CBCT showed sub‐millimeter shifts over treatment delivery. The average lateral shift was −0.07 mm with a standard deviation of 0.39 mm, vertical shift was −0.10 mm (±0.59 mm), and had a longitudinal shift of 0.07 mm (±0.43 mm). The average pitch, yaw, and roll for these patients were, respectively, −0.036 (±0.184), −0.109 (±0.230), and 0.027 (±0.172) degrees. Please note that the shifts were analyzed by the CT difference at two time points. Any motion during the treatment was not captured. In addition, the shifts in sub millimeter order reported by the fusion software may not depict the actual shifts during the treatment. There are limitations of registration algorithm to detect sub millimeter difference. Nevertheless, the results are negligibly small and consistent to the fact that once the vacuum suction between the customized mold and the upper hard palate is established, the patient is unable to make any movement. The non‐invasive Aktina device was able to provide the same level of immobilization as other invasive options.

### 3.2 Inter‐fraction set up error

The inter‐fraction reproducibility of a device can be different from intra‐fraction immobilization characteristics. In addition to the device design, user or therapist's skill and experience level can play an important factor. A good immobilization device should be able to minimize user dependence and be independent of skill and experience level of the therapist. In our study, Aktina and TALON did show significantly different characteristics in inter‐fraction reproducibility. While translation error can be corrected by couch shift, residual rotational error due to setup uncertainty cannot be corrected without a 6 DoF robotic couch. The uncorrected rotational error would potentially contribute to inaccuracy in radiation treatment delivery.

**Figure 5:**
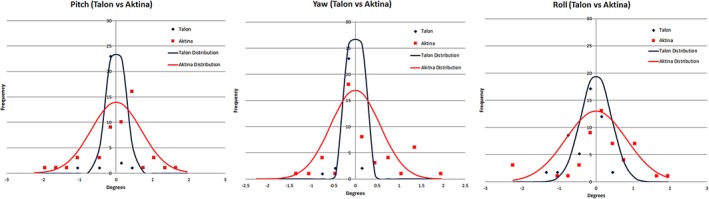
The distribution of residual rotational errors in pitch, yaw and roll directions. The curves were fitted assuming a Gaussian distribution. Please note the data points are histogram distribution of the measurement data, that is, the frequency of measurements in the error bin.

Figure 5 shows the distribution of residual rotational errors in pitch, yaw and roll for TALON and Aktina immobilization. Looking at pitch and yaw curves with TALON device, 25 out 28 treatments had essentially negligible residual pitch errors (23 between 0 to −0.3 degrees and 2 between 0 to 0.3 degrees). A patient could be set up more reproducibly with TALON with significantly smaller residual rotational errors than Aktina. The average residual error of TALON was −0.1 (±0.25), −0.08 (±0.16), −0.2 (±0.41) degrees, respectively, for pitch, yaw, and roll. That compared to 0.2 (±0.69), 0.34 (±0.56) and 0.35 (±0.82) for Aktina. Two‐sample Kolmogorov–Smirnov test was used to measure the significance of the distribution difference. For all three rotational dimensions, the differences were statistically significant with *P*‐values of 0.007656, 0.000353, 0.026248 in pitch, yaw, and roll, respectively. For both devices, roll direction had more set up uncertainty than the other two directions. That seems to be logical as it is more difficult to prevent patient from rotating the head in the roll direction.

### 3.3 Setup margin

**Figure 6:**
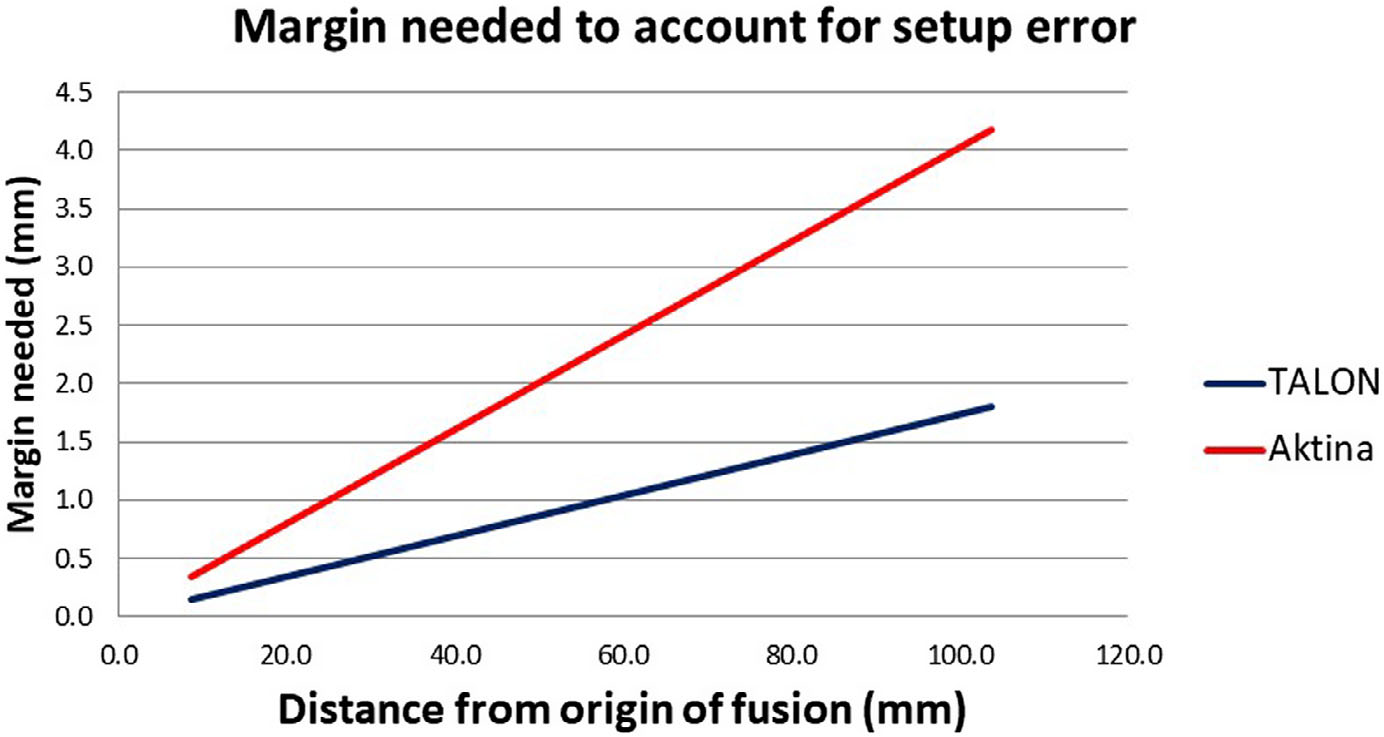
Additional GTV to PTV margin is needed to compensate for residual uncorrected rotational errors when lesion is away from the focus of image registration.

Varian, Elekta, and other conventional C‐Arm linacs can correct the yaw by couch rotation and Accuray Tomotherapy machine can correct the roll by automatic gantry rotation adjustment. However, without a 6 degree robotic couch, not all residual rotational errors can be corrected. Additional GTV to PTV margin may be necessary to compensate the set up inaccuracy to avoid geographical miss. The data of our study agreed reasonably to a Gaussian distribution with a mean centered approximately at zero (Fig. 5). In order to have 95% confidence interval, the additional margin needed varies with the distance of the tumor target to the image fusion center. For a single lesion, the registration can be focused at the center of the tumor. A small magnitude of rotation error should not significantly compromise the accuracy of the dose distribution. However, when multiple lesions are treated with a single plan, aligning one lesion would result in misalignment of another lesion due to residual rotational errors. Figure 6 illustrates additional margin needed when tumor target is away from the center of image fusion. When two lesions are separated by 10 cm and the center of the image registration is in between the two lesions, 1 mm and 2 mm additional margin is indicated for TALON immobilization and Aktina device, respectively.

## 4 DISCUSSION AND CONCLUSIONS

Over the years, many invasive and non‐invasive immobilization devices were created to minimize uncertainty in treatment delivery.^10^ Some examples of immobilization devices used over the years include rigid invasive SRS frame, TALON, surface tracking, plastic masks, and Aktina. To maximize patient comfort and compliance to treatment, a non‐invasive option is obviously preferred. In the recent years, even though frameless real‐time surface imaging‐guided radiosurgery facilitated by VisionRT™ (Vision RT Inc. Columbia, MD) or C‐Rad™ (C‐RAD Group, Uppsala, Sweden) has been reported to be feasible,^11^ it is our opinion that immobilization device are still necessary to assist in patient localization. In order to ensure the utmost precision in the delivery of radiation the patient must be accurately positioned, ensuring intra‐fraction motion and inter‐fraction errors are minimized. At our institution, Aktina has been used as an alternative to TALON. TALON was used for most cases where movement of the patient during and between treatments was detrimental. This study showed that while Aktina was not as accurate as TALON in inter‐fraction setup, the intra‐faction shifts were negligible at 0.5 ± 0.7 mm (lateral), 0.1 ± 0.9 mm (vertical), −0.5 ± 0.6 mm (longitudinal), −0.04 ± 0.18°(pitch), −0.1 ± 0.23°(yaw), and −0.03 ± 0.17°(roll). This, coupled with the non‐invasive nature of Aktina, makes Aktina a good alternative for patients needed cranial radiosurgery. The suction seal that attaches the mold to the upper hard palate ensures no shift in the patient during the treatment. One limitation of this study was that the intrafractional motion was evaluated by two CT scans before and after the treatment. Any patient motion during the treatment was not monitored. The inter‐fraction setup errors under Aktina immobilization could result from the user's lack of experience, for example, inaccuracy of placement of mouthpiece from day to day. Such errors were more common in Aktina than in TALON, as TALON provides a setup with extremely little room for user inconsistency. However, for patients that cannot tolerate the invasive nature of TALON, Aktina provides a valid alternative.

Kirkpatrick et al^12^ reported that brain radionecrosis is correlated with the GTV to PTV margin and recommended no more than 1 mm margin to be used in stereotactic radiosurgery. However, appropriate margin has to be applied to compensate for the accuracy of delivery. When the center of image registration is away from the lesion, additional GTV to PTV set up margin may be required to assure the radiation does not miss the target. One example of this scenario is when multiple lesions away from each other are treated in a single plan. It would be impossible to align perfectly to all the lesions if there are uncorrectable residual rotational errors. Additional margin may be required to ensure proper tumor coverage. Another alternative is to deliver the treatment with separate plans. Each plan includes only a single lesion or several lesions geometrically close so the image fusion can be focused on a limited region. A separate CBCT or MVCT can be acquired to minimize rotational errors thus no additional margin is needed. However, compared to a single plan, multiple separate plans come with significantly longer treatment times and other compromises. These include physics planning complexity and patient discomfort due to longer times immobilized on the couch.

A six degree of freedom robotic couch and other couch adapters can potentially correct all 6 dimensional setup errors. Gevaert et al.^13^ investigated the setup errors and intrafractional motion with and without 6 DoF couch. Forty patients immobilized with a BrainLab frameless mask system were monitored by BrainLAB ExacTrac stereoscopic X‐ray system. Without 6 DoF correction, the mean 3D setup error was 1.91 ± 1.25 mm and the mean 3D intrafractional motion was 0.58 ± 0.42 mm. They determined that the setup errors after 6 DoF correction were within action levels (1 mm for translations and 0.5 degree for rotations). The 6 DoF robotic couch can make all corrections generated by image registration automatically before treatment delivery. The setup is thus limited by image registration algorithm accuracy which is considered to be sub‐millimeter. Therefore, with a 6 DoF robotic couch, a single plan can be potentially used to treat multiple lesions that are far apart. This would significantly improve the treatment efficiency and reduce the time patient spends on the treatment couch. One thing to note is that not all LINAC manufactures currently support 6 DoF robotic couches. Optical surface image guidance has recently been adopted in intracranial SRS/SRT. The technology provides sub millimeter monitoring of the patient surface and can be useful to monitor intra‐fraction motion but has limited potential for reducing inter‐fraction setup error beyond the current immobilization devices.

## CONFLICT OF INTEREST

The authors declare no conflict of interests.
